# Mutation of Arabidopsis Copper-Containing Amine Oxidase Gene *AtCuAOδ* Alters Polyamines, Reduces Gibberellin Content and Affects Development

**DOI:** 10.3390/ijms21207789

**Published:** 2020-10-21

**Authors:** Basmah Alharbi, Julie D. Hunt, Simone Dimitrova, Natasha D. Spadafora, Alex P. Cort, Davide Colombo, Carsten T. Müller, Sandip A. Ghuge, Daniela Davoli, Alessandra Cona, Lorenzo Mariotti, Piero Picciarelli, Barend de Graaf, Hilary J. Rogers

**Affiliations:** 1 School of Biosciences, Cardiff University, Sir Martin Evans Building, Museum Avenue, Cardiff CF10 3AX, UK; b.alharbi@ut.edu.sa (B.A.); jhunt9953@gmail.com (J.D.H.); simon.dimitrova@nasekomo.life (S.D.); nspadafora@markes.com (N.D.S.); CortPA@cf.ac.uk (A.P.C.); davide@agridaeus.com (D.C.); mullerct@cf.ac.uk (C.T.M.); daniela.davoli91@gmail.com (D.D.); degraafb@cardiff.ac.uk (B.d.G.); 2 Department of Sciences, Università Roma Tre, Viale Marconi, 446, 00146 Roma, Italy; sandip.ghuge.biotech@gmail.com (S.A.G.); alessandra.cona@uniroma3.it (A.C.); 3 Department of Agriculture, Food and Environment, University of Pisa, Via Mariscoglio 34, 56124 Pisa, Italy; lorenzo.mariotti@unipi.it (L.M.); piero.picciarelli@unipi.it (P.P.)

**Keywords:** copper-containing amine oxidases, flowering, gibberellic acid, polyamines, putrescine, senescence

## Abstract

Polyamines (PAs) are essential metabolites in plants performing multiple functions during growth and development. Copper-containing amine oxidases (CuAOs) catalyse the catabolism of PAs and in *Arabidopsis thaliana* are encoded by a gene family. Two mutants of one gene family member, *AtCuAOδ*, showed delayed seed germination, leaf emergence, and flowering time. The height of the primary inflorescence shoot was reduced, and developmental leaf senescence was delayed. Siliques were significantly longer in mutant lines and contained more seeds. The phenotype of *AtCuAOδ* over-expressors was less affected. Before flowering, there was a significant increase in putrescine in *AtCuAOδ* mutant leaves compared to wild type (WT), while after flowering both spermidine and spermine concentrations were significantly higher than in WT leaves. The expression of GA (gibberellic acid) biosynthetic genes was repressed and the content of GA_1_, GA_7_, GA_8_, GA_9_, and GA_20_ was reduced in the mutants. The inhibitor of copper-containing amine oxidases, aminoguanidine hydrochloride, mimicked the effect of *AtCuAOδ* mutation on WT seed germination. Delayed germination, reduced shoot height, and delayed flowering in the mutants were rescued by GA_3_ treatment. These data strongly suggest *AtCuAOδ* is an important gene regulating PA homeostasis, and that a perturbation of PAs affects plant development through a reduction in GA biosynthesis.

## 1. Introduction

Polyamines (PAs) are low-molecular-weight organic cations. They are present at relatively high concentrations in all eukaryotic cells and interact with a range of macromolecules including DNA, RNA, proteins, and phospholipids to stabilise them. In plants, PAs are involved in a wide range of crucial cellular processes, for example, controlling cell division and elongation, plant growth and development, reproduction, and senescence [1]. Owing to their diverse role during plant growth and development, PAs can therefore justifiably be regarded as growth regulators [2].

The most common PAs in plants are the tri-amine spermidine (Spd), the tetra-amine spermine (Spm), and their di-amine precursor putrescine (Put). In most plant species, the first PA produced from the biosynthetic pathway of PAs is Put, which is an important intermediate precursor in the synthesis of the higher amines Spd and Spm. In Arabidopsis, Put forms indirectly from L-arginine by arginine decarboxylase (ADC) [3]. The conversion of Put to Spd and Spm requires the successive addition of aminopropyl moieties. Put is converted to Spd via sequential addition of an aminopropyl group generated from S-adenosylmethionine (SAM), via a reaction catalysed by SAM decarboxylase and by the action of spermidine synthase. Spd is then converted to Spm in a reaction catalysed by spermine synthase [4].

Because of the wide range of functions that PAs perform in plant cells, their homeostasis is critical and controlled via the regulation of their biosynthesis, conjugation, interconversion, and transport [1,5], as well as their catabolism. PA catabolism occurs through oxidative de-amination reactions at their primary or secondary amino groups by the action of amine oxidase enzymes [5,6,7]. Two classes of oxidative enzymes in plants are responsible for the degradation of PAs. Based on the cofactor involved, these are divided into copper-containing amine oxidases (CuAOs, EC 1.4.3.6), and flavin-containing PA oxidases (PAOs, E.C. 1.5.3.11). PAOs oxidize the higher PAs Spd and Spm at the secondary amino group [6,7]. In contrast, CuAOs are more specific for the di-amines Put and Cad, oxidizing them at the primary amino group. However, in Arabidopsis, three CuAO enzymes have been shown to also oxidize Spd but not Spm. These are apoplastic AtCuAOγ1 (At1g62810) and the peroxisomal AtCuAOα3 (At1g31710) and AtCuAOζ (At2g42490) [8,9]. The action of CuAOs on Put yields H_2_O_2_, ammonia (NH_4_^+^), and 4-aminobutyraldehyde, which then directly cyclizes to ∆^1^-pyrroline that is further converted to GABA by the influence of aldehyde dehydrogenase [7]. PA oxidation-derived H_2_O_2_ has been involved in cell wall lignification and maturation during development, wound-healing, and reinforcement of cell walls during pathogen infection [5,10]. Through its transamination and oxidization, GABA can enter the Krebs cycle, which ensures the recycling of carbon and nitrogen from PAs [11].

One role of PAs is in flowering: they appear to be involved in floral induction, differentiation, and in regulating fertility [12]. In both long- and short-day plants, foliar PAs increase as a response to photoperiodic induction, followed by their increase in the shoot apex where floral initiation eventually takes place [13,14]. In Arabidopsis, PAs are essential for flowering, and altering their levels can affect the transition to flowering [15]. Put and Spd are the prevalent PAs in Arabidopsis flowers, with Spd prevailing, in contrast to other organs where Spd and Put contents are lower. The application of Spd synthesis inhibitors that lower its endogenous levels completely inhibited bolting and flowering. Furthermore, treatment with Spd accelerated flowering of delayed-flowering mutant gigantea or WT Arabidopsis plants under short-day conditions where flowering is naturally late [15]. In contrast, the accumulation of Put, as a result of over-expressing the PA biosynthetic gene *ADC2*, delayed flowering time [16]. The delay was attributed to a decrease in the contents of bioactive GAs as a result of the down-regulation of the expression of two GA biosynthetic genes *GA 20-oxidase* and *GA 3-oxidase*. The phenotype was rescued by exogenously applied GA_3_ [16], indicating that increased Put did not affect GA signalling.

In Arabidopsis, copper-containing amine oxidase genes (CuAOs) form a gene family of ten members with different patterns of expression. However, of these, only eight are thought to encode functional enzymes [9]. *AtCuAOδ* (At4g12290) encodes an enzyme predicted to localise in plastids [17], although it has also been identified in the rosette leaf vacuole proteome [18]. The expression of *AtCuAOδ* appears to be primarily in mature seeds and senescent leaves but is also expressed highly in the hypocotyl, guard cells, sepals and stigma [19,20]. Expression of *AtCuAOδ* is also up-regulated by pathogens and Pep2, a peptide which activates the innate immune response [21], as well as a range of abiotic stresses and ABA. It is thought to play an important role in stomatal closure [20].

Here, data are presented on the perturbation of *AtCuAOδ* expression. Mutants show a range of phenotypic changes as well as a change in PA and GA content, indicating that copper-containing amine oxidase genes are important in whole plant development. Moreover, our results indicate that *AtCuAOδ* is an important gene in regulating PA homeostasis and that a perturbation of PAs affects plant development through the reduction in GA biosynthesis.

## 2. Results

### 2.1. Perturbation of AtCuAOδ Expression Has a Weak but Significant Effect on Leaf Senescence

**Figure 1:**
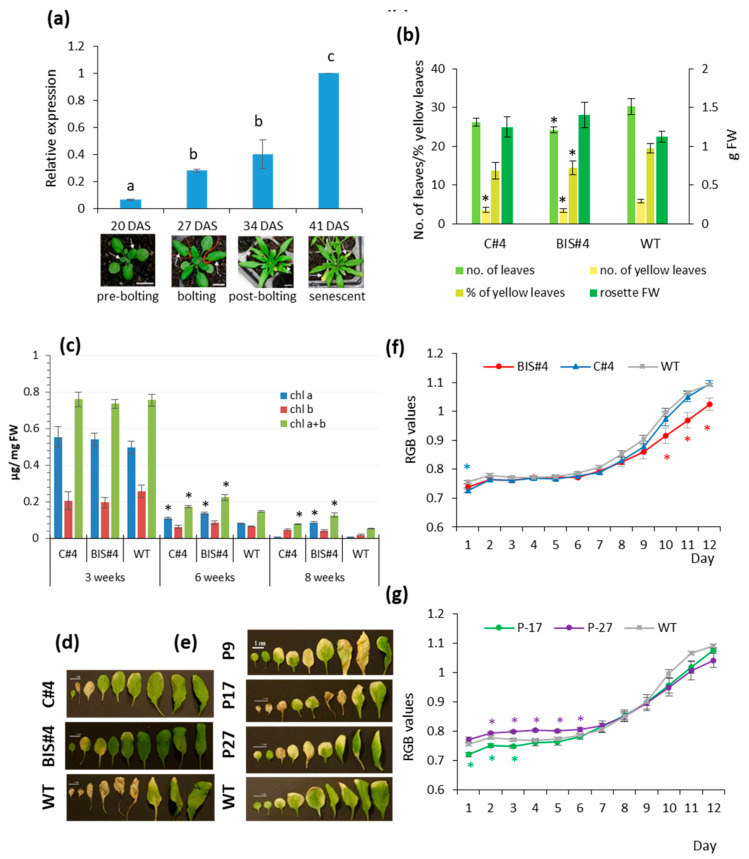
The expression of *AtCuAOδ* and the effects of its perturbation on senescence: (**a**) gene expression in leaf 5 and 6 of rosettes during leaf development and senescence (n = 3, ± SE); (**b**) total number of leaves, number of yellow leaves, % of yellow leaves, and rosette fresh weight after 8 weeks growth of the two mutants C#4 and BIS#4 (n = 8, ± SE); (**c**) chlorophyll levels in leaves no. 5 and 6 of mutants C#4 and Bis#4 at three different stages (± SE, n = 6); (**d**) leaf yellowing of the nine oldest leaves (oldest to youngest, left to right) from 8-week-old rosettes (scale bar = 1 cm) of the two mutants C#4 and BIS#4; (**e**) over-expressor lines P9, P17, and P27; (**f**) yellowing as measured by changes in RGB (increased yellowing) during dark-induced senescence in C#4 and BIS#4; (**g**) over-expressor lines P17 and P27 (n = 9, ±SE). Significant differences in means are indicated by * at *p* ≤ 0.05 based on a *T*-test; different lettering is based on a one-way ANOVA followed by a Tukey’s test; DAS is days after sowing.

Expression of AtCuAOδ rose significantly during rosette leaf development in WT Arabidopsis leaves (Figure 1a), increasing steadily through bolting and into early senescence. This suggests that this gene may have a role during leaf senescence. In agreement with this hypothesis, two insertional mutants of AtCuAOδ (BIS#4 and C#4), in which expression of the full-length transcript is abolished (Supplementary Figure S1), both showed a significantly lower number of yellowing leaves after 6–8 weeks of growth compared to WT (Figure 1b,d), although the % of yellow leaves was only statistically significant for line C#4. Both mutants also showed higher chlorophyll levels after 6–8 weeks of growth compared to WT (Figure 1c). Verification of mutant lines using three different combinations of primers (upstream, flanking, and downstream; Supplementary Figure S1) showed that neither mutant, C#4 or BIS#4, produced transcripts flanking the T-DNA insertion site, while a product was obtained upstream of the insertion point. It is assumed therefore that although truncated transcripts are produced, the gene is essentially knocked out by both insertions and no functional AtCuAOδ protein can be produced by either of them. However, rosette fresh weight and the total number of leaves were not consistently affected by the mutation (Figure 1b). To explore further the role of this gene, three independent over-expression lines (P9, P17, and P27) were tested, in which expression of the AtCuAOδ open reading frame fused to a His-Tag is driven by the CaMV 35S promoter. Over-expression of AtCuAOδ had less effect on natural senescence than insertional mutation (Supplementary Figure S3); although leaves appeared to senesce earlier (Figure 1e), the only significant difference was a reduced number of leaves in one over-expression line (Supplementary Figure S3).

Dark-induced senescence in detached rosettes was also significantly delayed in one of the mutant lines (BIS#4), although senescence progression appeared to be unaffected in line C#4 (Figure 1f). Conversely, senescence was slightly accelerated in over-expressor line P27 at the start of the dark period, but in line P17 there was a small but significant delay (Figure 1g).

Thus, overall, the knockout of AtCuAOδ seems to result in a mild but significant delay in senescence, while over-expression of this gene has an even weaker and less consistent effect on accelerating senescence.

### 2.2. Bolting and Flowering Time Are Affected by Perturbation of AtCuAOδ Expression

**Figure 2:**
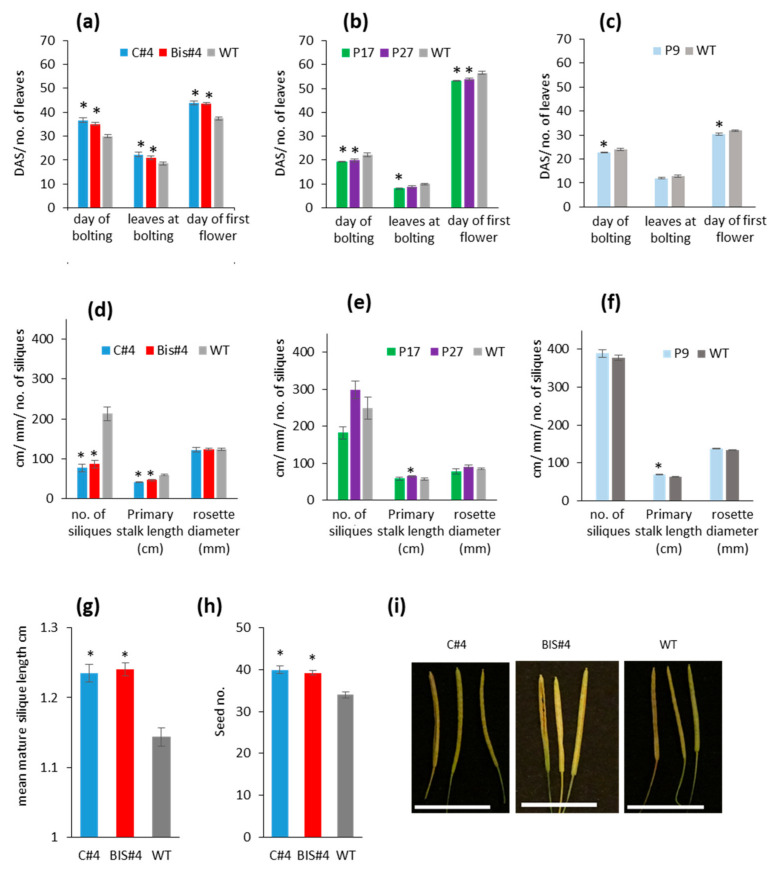
Perturbation of *AtCuAOδ* expression affects flowering time, stalk height, silique number, and their length: day of bolting and flowering and number of leaves at bolting in (**a**) mutants C#4 and BIS#4 (n = 8); (**b**) over-expressor lines P17 and P27; (**c**) line P9 (n = 8). Also shown are the primary stalk length, number of siliques, and rosette diameter after 8 weeks growth of (**d**) two mutants C#4 and BIS#4 (n = 8); (**e**) over-expressor lines P17 and P27; (**f**) line P9 (n = 8); (**g**) mean mature silique length (n = 100); (**h**) seed number per silique (n = 100 siliques); and (**i**) silique images (scale bar = 1cm) of the two mutants C#4 and BIS#4 siliques; means ± SE, significant differences are indicated by * at *p* ≤ 0.05 based on a Student *t*-test where data were normally distributed or Mann-Whitney test where data were not.

Plants from *AtCuAOδ* BIS#4 and C#4 mutant lines bolted and flowered significantly later, by 5–6 days and 7 days, respectively, and produced a greater number of leaves before bolting relative to WT (Figure 2a). The day of bolting and of the first flower produced by the three over-expressor lines was conversely significantly earlier (2–3 days and 3–4 days respectively) than in WT plants (Figure 2b,c). Primary inflorescence length was significantly shorter in both mutants after 8 weeks’ growth compared to WT (Figure 2d) and was slightly but significantly longer than WT in two of the over-expressor lines (P27 and P9) but not P17 (Figure 2e,f). However, rosette diameter was not affected by the perturbation of *AtCuAOδ* expression. Siliques were significantly longer than WT in both mutants and contained more seeds (Figure 2g–i); however, the total number of siliques produced after 8 weeks’ growth was significantly lower in the mutant lines (Figure 2d). Mutation of *AtCuAOδ* had no effect, however, on the rate of silique production relative to WT plants, as there was no significant difference in the number of siliques produced over one week starting from the day of formation of the first silique (Supplementary Figure S3c). The silique number was not significantly affected in the over-expressor lines (Figure 2e,f).

### 2.3. Mutation of AtCuAOδ Results in Delayed Seed Germination and Leaf Emergence While Over-Expression Has Fewer Effects

**Figure 3:**
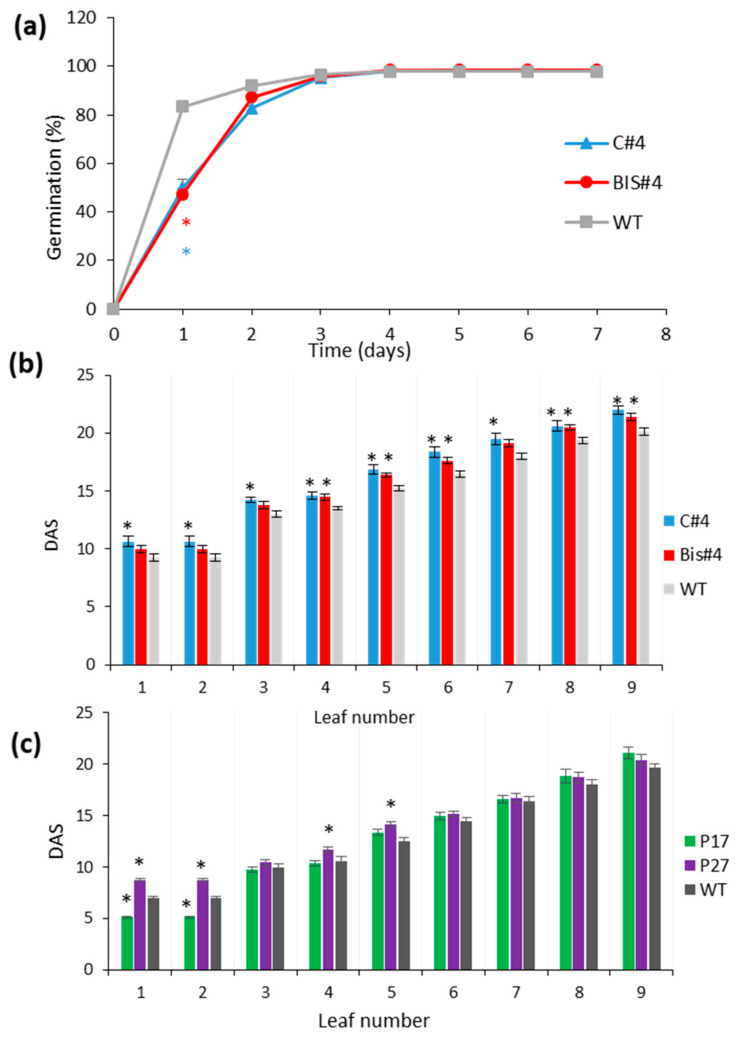
The effect of *AtCuAOδ* mutation on germination and leaf emergence mutant lines (C#4 and BIS#4) and over-expressor lines P17 and P27 compared to WT: (**a**) the germination rate of mutants C#4 and BIS#4 (n = 5; 30 seeds/replicate); (**b**) leaf emergence in mutant lines C#4 and BIS#4 (n = 8); (**c**) leaf emergence in over-expressor lines P17 and P27 (n = 8–16). Significant differences in means are indicated by * at *p* ≤ 0.05 based on a Student *t*-test. DAS is days after sowing, mean ± SE.

Radical protrusion in the two T-DNA insertion lines of *AtCuAOδ*, C#4 and BIS#4 was significantly delayed during seed germination (Figure 3a) compared to WT 1 day after sowing, but by 3 days all seeds had germinated. In contrast, germination in all three over-expressor lines (P9, P17, and P27) was not significantly different from WT (Supplementary Figure S4). Leaf emergence was also affected in the insertional mutants: the emergence of leaves 1–9 was delayed significantly in the C#4 mutant line, and the emergence of leaves 4, 5, 6, 8, and 9 was also delayed in BIS#4 compared with WT, by about 2 days (Figure 3b). The delay in leaf emergence in the mutant lines is likely linked to the delayed germination. Again, in the over-expressor lines effects were less clear, although the emergence of leaves 1 and 2 was anticipated in lines P17 and P27 and leaves 4 and 5 in line P27 (Figure 3c). There were even fewer significant differences in line P9 (Supplementary Figure S4).

### 2.4. PA Content Is Perturbed in AtCuAOδ Mutants and Treatment of WT with the Copper-Containing Amine Oxidase Inhibitor, Aminoguanidine Hydrochloride, Mimics the AtCuAOδ Mutant Germination Phenotype

**Figure 4:**
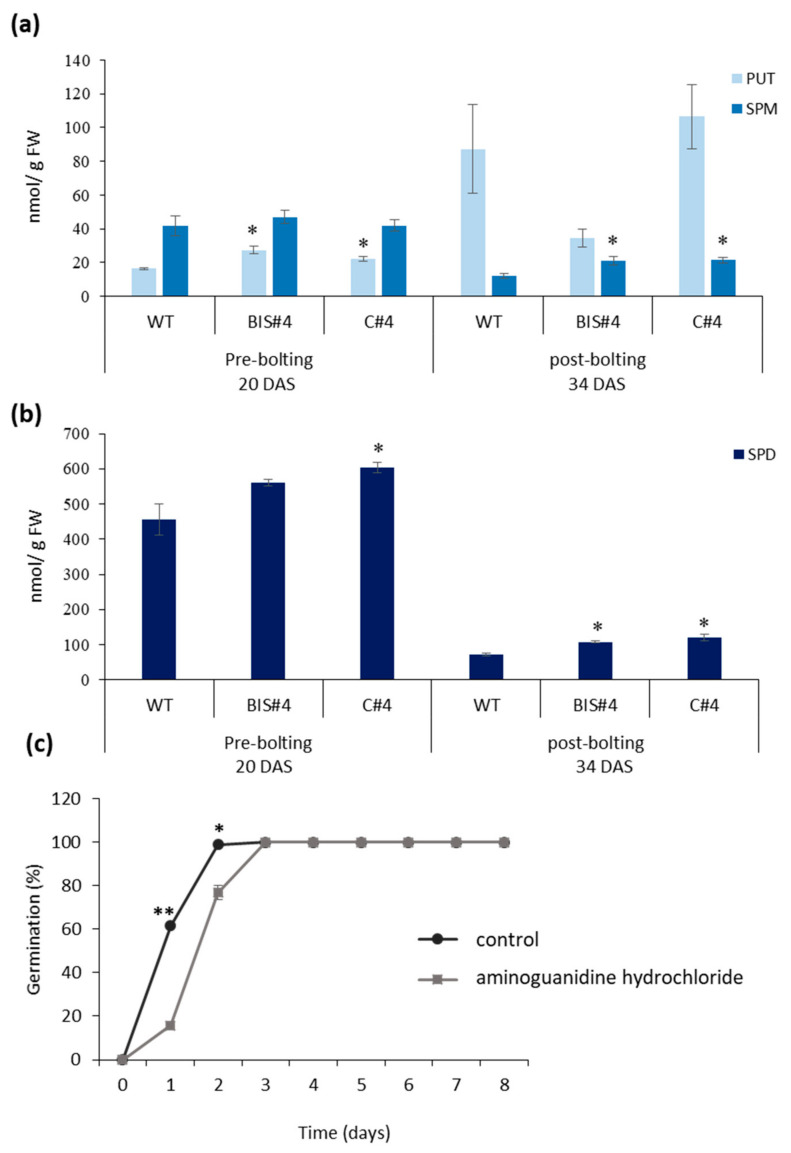
Free polyamine contents in leaves 5 and 6 of the two mutant lines (C#4 and BIS#4) and WT plants pre- and post-bolting and the effect of aminoguanidine hydrochloride on germination of WT. (**a**) The content of the di-amine Put and the tetra-amine Spm, (**b**) the content of the tri-amine Spd, (n = 6); (**c**) seed germination of WT on water agar medium containing 1 mM aminoguanidine hydrochloride, data from three biological replicates (~60 seeds/replicate); Asterisks indicate values significantly different from wild type (WT) or control at *p* ≤ 0.05 based on a Student *t*-test where data were normally distributed or Mann-Whitney test where data were not. Mean values ± SE.

Given that phenotypic effects were more consistent and significant in mutants of *AtCuAOδ* compared to over-expressors, experiments focused on investigating the possible underlying mechanisms responsible for the mutant phenotype. To test whether the phenotypic effects could be ascribed to a perturbance of PA metabolism, contents of putrescine, spermidine and spermine were analysed before and after bolting in leaves 5 and 6 of 20- and 34-day-old mutant and WT plants (Figure 4a). There was a 1.7- and 1.36-fold increase in the free Put content in leaves of BIS#4 and C#4, respectively, compared to the WT pre-bolting. No differences in the content of the free form of the tetra-amine Spm were observed pre-bolting between mutants and WT leaves. Put content increased in both mutants and WT during reproduction compared with the vegetative stage, but this increase was not significantly different among the three tested lines. However, although Spm content in leaves declined post-bolting relative to the vegetative stage, its content in leaves of both mutants was significantly higher and almost double (1.75-fold) that in WT leaves. Spd content in its free form was higher pre-bolting than post-bolting in both mutants and WT. Pre-bolting, Spd was higher than WT in both leaves of both mutants; however, this difference was only significant in leaves of C#4 compared with WT (Figure 4b). Free Spd decreased in leaves of both mutants and WT post-bolting relative to their levels in the pre-bolting stage. However, free Spd content was significantly higher in leaves of mutant plants compared with WT showing a ~1.5- and ~1.7-fold increase in BIS#4 and C#4 leaves, respectively, compared to WT leaves at the same stage.

To verify whether the *AtCuAOδ* mutant phenotype could be explained by a reduction in copper-containing amine oxidase activity, an inhibitor was tested on WT for its ability to mimic the delayed germination phenotype of the mutants (Figure 4c). The inhibitor aminoguanidine hydrochloride affected germination % significantly after 1 and 2 days, but by 3 days germination was restored to the control, indicating that there was a delay rather than complete inhibition of germination.

### 2.5. Treatment with GA Reverses the Effects of AtCuAOδ Mutation on Seed Germination, Bolting Time and Inflorescence Stem Length

**Figure 5:**
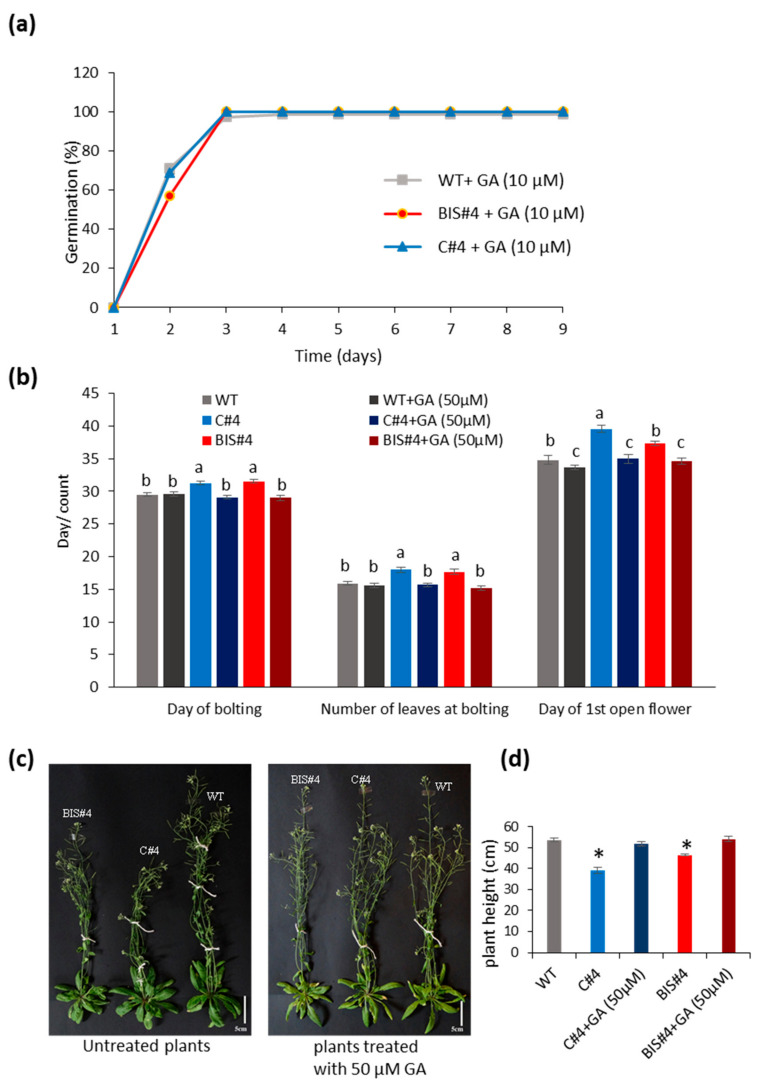
The effect of exogenous application of GA_3_ on *AtCuAOδ* mutant lines (C#4 and BIS#4) and wild type plants: (**a**) % germination on water agar medium containing 10μM GA_3_; (**b**) day of bolting, number of leaves at bolting, and day of 1st flower opening with and without application of 50 μM GA3 (n = 12); (**c**,**d**) stalk length with and without application of 50 μM GA_3_; scale bars = 5 cm. Error bars represent the standard error of the mean. Letters indicate a statistically significant difference between treatments within each character measured using one-way ANOVA (*p* ≤ 0.05). Asterisks indicate a significant difference from WT at *p* ≤ 0.05 based on a Student *t*-test. The experiment was repeated twice with similar results.

To test whether GA could be involved in the mutant phenotype, exogenous GA was applied during seed germination and before flowering. Treatment of germinating seeds with GA abolished the delay in germination seen in both C#4 and BIS#4 mutants and reverted phenotype to WT (Figure 5a). Likewise, the delayed bolting and flowering and increased number of leaves at bolting seen in the two mutants (Figure 2a) were reversed when the two mutant lines were sprayed with 50 μM GA_3_ as compared to treated with water (Figure 5b). The reduced inflorescence stem length seen in the two mutants (Figure 2d) was also restored to WT (Figure 5c,d). At this concentration of GA_3_, WT development was not affected.

### 2.6. Endogenous Levels of GAs Are Reduced in AtCuAOδ Mutants But Auxin Level Is Not Consistently Reduced

**Figure 6:**
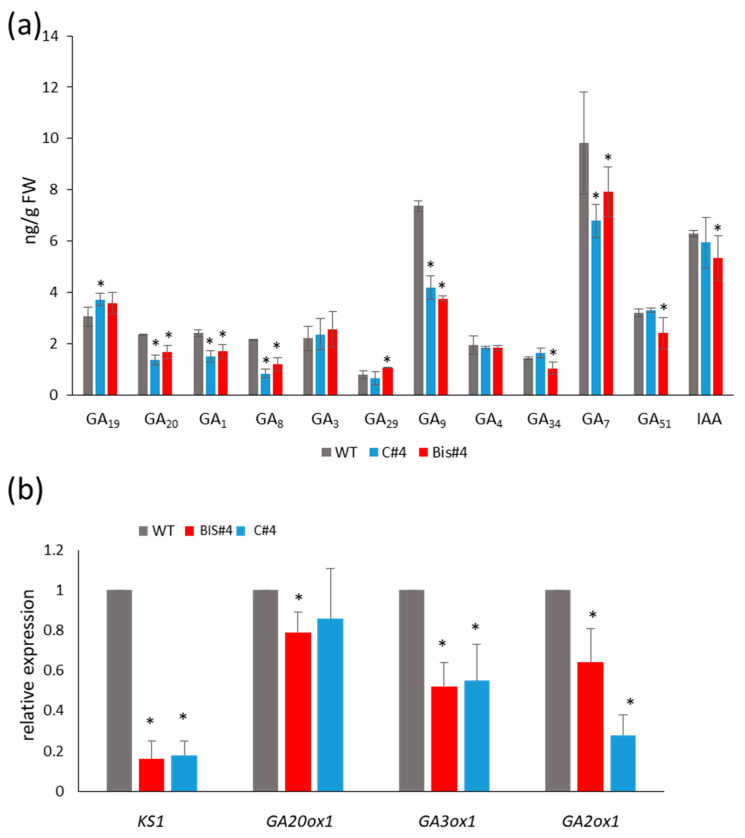
GA content and expression of GA biosynthetic genes in *AtCuAOδ* mutants compared to WT. (**a**) Quantitative analysis of endogenous gibberellins by GC-MS/MS extracted from 2-week-old seedlings. (**b**) Real-time PCR of GA biosynthetic genes, *KS1*, *GA20ox-1*, *GA3ox-1*, and *GA2-ox-1*. n = 3; means ± SE; asterisks indicate significant differences to WT (*p* ≤ 0.05), based on a Student *t*-test.

Given the reversal of the mutant phenotypes with the application of exogenous GA, endogenous GAs were analysed to assess whether the mutant phenotype could be explained by a reduction in endogenous GA levels. The endogenous GA content of the 13-non hydroxylation (GA_9_, GA_4_, GA_34_, GA_7_, and GA_51_) and the early 13-hydroxylation (GA_19_, GA _20_, GA_29_, GA_1_, GA_8_ and GA_3_) pathways were quantified in Arabidopsis (WT) and in *AtCuAOδ* mutant (lines C#4 and BIS#4) pre-bolting seedlings (Figure 6a). Profiling of the GA content revealed higher metabolic flow in WT plants than in *AtCuAOδ* mutant lines. The endogenous levels of precursor GA_20_, GA_9_, and catabolite GA_8_ were significantly higher in WT than in both mutant lines, while GA_29_, GA_51_, and GA_34_ were only reduced in one or other mutant. There was a slight increase in GA_19_ but only in C#4 and not in Bis#4. The content of bioactive GA_1_ and GA_7_ was reduced, however, in both mutant lines. Auxin (IAA) content was reduced in the Bis#4 mutant but not in C#4 compared to WT.

### 2.7. Expression of GA Biosynthetic Genes Is Reduced in AtCuAOδ Mutants

Since the metabolic flow of GAs was reduced in the mutant seedlings, the expression of key genes involved in GA biosynthesis was analysed by real-time PCR in two-week-old seedlings of C#4, BIS#4, and WT (Figure 6b). *GA20ox1*, *GA3ox1,* and *GA2ox1* were selected as the members of their respective gene families that are expressed in vegetative tissues. The expression of *KS1* was dramatically reduced in both mutants by over 5-fold compared to WT. The expression of *GA3ox1* and *GA2ox1* was also consistently reduced in the *AtCuAOδ* mutants. Expression of *GA20ox1* was also reduced in the mutants but the reduction was only significant in mutant line BIS#4.

## 3. Discussion

The expression of *AtCuAOδ* increases dramatically during leaf senescence, in agreement with database microarrays that show enhanced expression in cauline and senescent leaves [19]. These data might suggest that the most important effects of *AtCuAOδ* would be on senescence, perhaps involved in the remobilisation of nitrogen reserves, as has been previously suggested for PA catabolic genes [7]. However, knocking out this gene delays germination, leaf development, and flowering, with only a mild effect on senescence, and over-expression has more limited effects, with consistent effects only on bolting and flowering time. Thus, perturbation of *AtCuAOδ* expression clearly affects different stages of plant development as well as, to a minor extent, senescence. Natural senescence in Arabidopsis is not directly linked to flowering time [22], but it is linked to seed production and the loss of meristem activity. Hence, the delayed rosette senescence could be attributed entirely to a delay in seed formation rather than a direct effect on senescence. Dark-induced leaf senescence shares downstream pathways with developmental senescence [23], but upstream regulation differs [23,24], and it is independent of effects relating to flowering time. Effects of dark-induced rosette senescence were not consistent between the two *AtCuAOδ* insertion lines, supporting the hypothesis that despite its expression pattern, the major effects of perturbing this gene occur pre-bolting.

The effects of PAs in regulating plant growth and development are mediated by the regulation of cellular PA levels [8]. Hence, an obvious hypothesis for the phenotypic effects in both mutant *AtCuAOδ* lines was that they were caused by a change in the concentration of free PAs, and specifically, Put, as this is the substrate of most characterized AtCuAOs [25]. The concentrations of different PAs found in WT leaves during vegetative and reproductive stages were similar to those found previously [26], and the increase in Put in the mutants pre-bolting is consistent with a reduction in CuAO activity and hence an accumulation of substrate. Indeed, treatment of WT Arabidopsis seeds with the inhibitor of copper-containing amine oxidase activity, aminoguanidine hydrochloride, mimicked the loss of *AtCuAOδ* in a delay in germination. This is consistent with the accumulation of the *AtCuAOδ* transcript in mature dry seeds, presumably required for translation into active enzyme during germination and early seedling growth. This suggests that effects seen in *AtCuAOδ* mutants may indeed be due to a reduction in CuAO enzymatic activity, although other activities cannot be excluded. There was no significant Put increase in the mutants post-bolting, despite the higher expression of this gene post-bolting in WT. There was, however, a significant increase in both Spm and Spd post-bolting in the *AtCuAOδ* mutants. This could be a consequence of the increased Put being converted to Spd and Spm via spermidine and spermine synthase respectively [7], thus restoring the post-bolting PA balance. The increases in Spm and Spd noted in the *AtCuAOδ* mutants contrast with the null effect on these metabolites of increasing Put through over-expression of the *ADC2* gene [16]. This may be because the very high Put increase elicited by *ADC2* over-expression results in a toxic effect, whereas the homeostatic mechanisms are able to respond to the more moderate Put increase in the *AtCuAOδ* mutants. Although mutation of *AtCuAOδ* represses only one gene involved in Put catabolism, it was enough to change Put levels pre-bolting. This indicates that *AtCuAOδ* plays an important role in PA homeostasis. Phenotypic changes seen in the mutants indicate furthermore that expression of *AtCuAOδ* pre-bolting is required for the correct timing of leaf development and the initiation of flowering.

The phenotypic effects of *AtCuAOδ* mutants resembled those seen when Put levels were artificially increased by over-expression of *ADC2* in transgenic plants [16], including a reduction in plant height and an increase in the number of leaves at bolting. A reduction in GA synthesis or perception delays flowering senescence in Arabidopsis [27]. Hence, Alcazar et al. [16] concluded that the effects of increased Put might be mediated by a reduction in GA biosynthesis, and indeed demonstrated that GA levels were reduced in *ADC2* over-expressors and there was also reduced expression of GA biosynthetic genes. A GA effect is also consistent with the *AtCuAOδ* mutant phenotype, including the delay in germination, which is also a known effect of a reduction in GAs [28]. Furthermore, the phenotype of the *AtCuAOδ* mutants was restored by exogenous GA_3_ application, a further indication that the phenotype is not caused by GA responsiveness but rather by the inhibition of GA biosynthesis.

**Figure 7:**
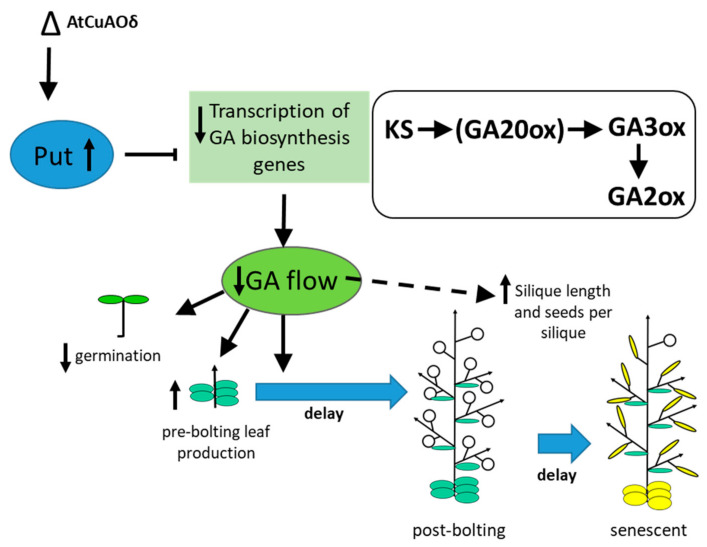
Diagram showing the effects of *AtCuAOδ* mutation on plant growth regulator balance and changes in development. The loss of *AtCuAOδ* function results in a premature increase in Put. This in turn down-regulates the expression of GA biosynthetic genes (specifically *KS*, *GA20ox* (less so), *GA2ox* and *GA3ox*) resulting in a reduction of the flow through the gibberellin pathway. This results in a delay in germination and bolting (black arrows). Delayed seed production results in delayed rosette senescence. Effects on silique length and number of seeds per silique may be directly mediated by the reduction in GAs or may occur via a different mechanism.

In agreement with Alcazar et al. [16], the increased Put in *AtCuAOδ* mutants resulted in significantly reduced amounts of GA_9_ and GA_20_. Both GA_7_ and GA_1_ were also significantly reduced in both mutant lines. However, unlike effects seen with *ADC2* over-expression, GA_4_ amounts in *AtCuAOδ* mutants were no different from WT. The key bioactive GAs in plants are GA_1_, GA_3_, GA_4_, and GA_7_ [29]. In the *AtCuAOδ* mutants the pattern of GA reduction indicates a reduction in bioactive-GA precursor biosynthesis since GA_9_, and GA_20_ are precursors of GA_4_, and GA_1_ respectively, reflecting, therefore, a reduction of the flow through the gibberellin pathway. This is also consistent with the reduction in the expression of *KS1* which catalyses the formation of ent-kaurene, the precursor to all the GAs. However, the reduction in *KS1* expression was not found when *ADC2* was over-expressed [16]. The reduction in the expression of *GA2ox1* in *AtCuAOδ* mutants is consistent with the reduction in GA_8_, but again was not found as a result of *ADC2* over-expression [16]. However, the significant reduction in *GA20ox* expression in at least one of the *AtCuAOδ* mutants is consistent with the reduction in GA_9_ and GA_20_ and was also found in the *ADC2* over-expressor lines [16]. Likewise, the reduction in *GA3ox1* expression seen in both mutants here is consistent with the reduction in GA_1_ and GA_7_ in the *AtCuAOδ* mutants and was also found in the *ADC2* over-expressors. Thus, the overall pattern is broadly consistent with previous findings: an increase in Put pre-bolting results in an inhibition of flow through the gibberellin pathway, a reduction in the transcription of GA biosynthetic genes (Figure 7), and results in phenotypic effects associated with reduced GA signalling. Importantly these data show that this effect is also seen in mutants of a catabolic PA enzyme. The precise pattern differs between *ADC2* over-expressors and *AtCuAOδ* mutants, perhaps due to the complex regulation of GA biosynthesis [29,30] and the very different levels of Put.

Cross talk between PAs and auxin has been discovered in the *ACL5* gene which encodes a thermospermine synthase involved in polar auxin transport [31,32]. PAs have also been linked to ethylene biosynthesis through the shared S-adenosyl-methionine decarboxylase activity required for their biosynthesis. Moreover, *BUD2*, which encodes a SAM decarboxylase needed for PA biosynthesis, is auxin-regulated [33]. Previous work has also indicated a link between GA and auxin [34], with auxin responsible for maintaining levels of *GA3ox* gene expression. Furthermore, in tomato fruit, a rise in IAA correlated with an increase in *GA20ox* expression while reducing the expression of *GA2ox* [35]. Although these are clearly different systems to Arabidopsis, a potential link between an increase in Put and a reduction in expression of GA biosynthesis genes in *AtCuAOδ* mutants could be mediated by a change in auxin levels. However, the concentration of IAA in *AtCuAOδ* mutants was not different from WT in both mutants. This indicates that the cross talk is unlikely to be mediated directly via a change in auxin concentration, although further analyses would be required to establish whether the increase in Put might affect auxin transport.

Although many of the effects of AtCuAOδ mutation appear to be pre-bolting, there were also significant effects on silique length and seeds per silique. *Ga20ox1* mutants did not show delayed flowering, and seed number per silique was not affected but they had slightly longer siliques than WT [36]. However, mutation of both *Ga20ox1* and *Ga20ox2* resulted in shorter siliques with fewer seeds per silique. *GA2ox* mutants also had shorter siliques with fewer seeds [37]. These results contrast with the phenotype of *AtCuAOδ* mutants that have longer siliques with more seeds. Thus, the effect on seed production of perturbing GA biosynthesis and interconversion appears to be quite complex. It is therefore not possible to be certain whether the effects on siliques resulting from *AtCuAOδ* mutation are directly elicited by a change in GA content or whether the effect is indirect.

## 4. Materials and Methods

### 4.1. AtCuAOδ Over-Expressors and Insertional Mutants

Two homozygous mutant lines in Arabidopsis thaliana (L.) Heynh ecotype Columbia (Col-0) with a T-DNA insertion in the first exon of the *AtCuAOδ* (At4g12290) gene were obtained from the lab of Prof A. Cona, and were originally from the Nottingham Arabidopsis Stock Centre: SALK_072954.55.00.x (also named Atcuaoδ.1 [20], here referred to as line C#4) and GK_011C04-013046 (also named Atcuaoδ.2 [20], here referred to as line BIS#4). The positions of the insertions were verified by PCR (Supplementary Figure S1 and Table S1).

The three independently selected transgenic lines, P9, P17, and P27 over-expressing the copper amine oxidase gene *AtCuAOδ* (At4g12290) were previously described [20], with the expression of *AtCuAOδ* driven by the 35SCaMVpromoter. Western blot analysis indicated expression levels were P27 > P9 [20]. The expression of line 27, not previously published, was verified by Western blotting and real-time PCR as previously described [20] (Supplementary Figure S2).

### 4.2. Plant Growth Conditions and Germination Assays

Homozygous over-expressor and T-DNA insertion *AtCuAOδ* lines were grown alongside WT in soil (sterilized sand and commercial multi-purpose compost, 1:3, *v*/*v*) in a Sanyo-Fitotron, (Loughborough, UK) growth chamber. Plants were grown under long days (16 h light) for analysis of flowering time, leaf development, silique length, and developmental senescence, or short days (8 h light) for the dark-induced senescence experiment, at 120–140 µmol m^−2^ s^−1^ at 21 °C in 9 cm pots. For growth on sterile media, seeds were surface sterilised then sown onto Murashige and Skoog (4.708 g/L) basal salt (Duchefa Biochemie, Haarlem, The Netherlands), in 9 cm Petri dishes supplemented with 1% DifcoTM agar and 1% (*w*/*v*) sucrose, pH 5.5–5.7, supplemented with Kan at 50 µg/mL for heterozygous over-expression lines or without for WT seeds. For all experiments, seeds were harvested at approximately the same time from individual plants grown in identical environmental conditions.

For germination assays, seeds of comparable age were plated as stated above on water agar medium (1.5%) and germination was monitored every 24 h. Germination was scored as radicle emergence from the testa of the seed.

### 4.3. Treatments with GA and Aminoguanidine Hydrochloride

Whole plants were treated with GA_3_: a GA_3_ stock solution (2.89 mM in ethanol) was diluted into 500 mL of a 0.02% Tween-20 solution to a final concentration of 50 μM GA_3_. Plant rosettes were sprayed from the 6–7 leaf stage on six alternate days until the onset of bolting, spraying until droplets covered all leaves. Control plants were sprayed with a solution containing an equivalent concentration of ethanol (1.73%) and containing 0.02% Tween-20. The concentration 50 µM/mL of GA_3_ was chosen based on Devaiah et al. (2009) [38]; a higher concentration of 100 µM/mL was tested and gave severe abnormalities in plant growth. Seed germination in the presence of GA_3_ was performed by including 10 μM GA_3_ in the 1.5% water agar medium based on the concentration used by Reference [16]. Seed germination assays in the presence of a PA oxidase inhibitor were performed on 1.5% agar medium containing 1 mM aminoguanidine (AG) as per Shevyakova et al. [39].

### 4.4. Dark Induced Senescence and Analysis of Chlorophyll

Plants were grown in soil under short-day conditions as described above for 30 days then rosettes were rinsed briefly in sterile water and placed on a 9 cm wet filter paper in the lid of a Petri dish. Dishes were incubated at 22 °C in complete darkness inside a thick-walled plastic box and photographed daily until rosettes turned completely yellow. Images were analysed using Image J (open source software) to obtain the RGB score and normalized using a white-background reference point.

For chlorophyll analysis, leaves 5 and 6 from mutant and wild type rosettes grown in soil were sampled (6 leaves/time point) and extractions carried out according to Reference [40] via extraction in methanol and measurement of absorbance using a microplate reader (Tecan, Männedorf, Switzerland).

### 4.5. PA Analysis

PAs were extracted according to Reference [41]: three biological replicates of 150–200 mg of leaves were ground to a fine powder in liquid nitrogen and homogenized in 5% (*v*/*v*) cold perchloric acid (PCA; Fisher Scientific, Loughborough, UK) at a ratio of 100 mg fresh weight/300 µL PCA in an ice bath. After 30 min of incubation on ice, homogenates were centrifuged at 17,000× *g*, 4 °C, for 25 min in a Heraeus Fresco 17 centrifuge (Thermo scientific, Waltham, MA, USA). PAs were dansylated with 200 μL of perchloric acid extracts mixed with 200 μL of saturated sodium carbonate and 400 μL of dansyl chloride (5 mg/mL in acetone, Sigma Aldrich, Poole, UK) with the addition of 40 μL of 0.05 mM 1,7 diamino heptane (DIA; Sigma Aldrich, Poole, UK) as an internal standard. After vortexing for 30 s they were incubated in the dark at 70 °C for 10 min. Reactions were stopped with 100 μL of 100 mg/mL proline and incubated in the dark for 30 min at room temperature. Dansylated PAs were extracted with toluene and then evaporated under a stream of nitrogen gas. Dry PAs were re-suspended in 400 μL of acetonitrile and filtered with 0.45 μm PVDF membrane prior to injection of 20 μL of the sample into a reversed-phase HPLC (Thermo scientific, Waltham, MA USA). The derivatised PAs were separated on a Phenomenex (Macclesfield, UK) Max-RP column (250 mm × 4.6 mm with Phenomenex (Macclesfield, UK) C18 security guard) at room temperature (22 °C). Elution was placed in a gradient of acetonitrile and water for 15 min. Initial conditions were 70% acetonitrile and 30% water at a flow-rate of 1.5 mL/min. The mixture was pumped for 4 min; then, the acetonitrile concentration was increased to 100% and was kept constant at this concentration for another 4 min, and finally returned to the initial conditions. Areas of each sample peak were integrated and compared to those of the standards of known concentration.

### 4.6. Real Time PCR

RNA was isolated from frozen material (three biological replicates) using an RNeasy Plant Mini kit (QIAGEN, Manchester, UK) or Tri reagent (Sigma Aldrich, Poole, UK)as described by the manufacturer. Genomic DNA was removed from RNA samples using RQ1 RNase-(Promega, Madison, WI, USA) and success was verified by PCR using 18S rRNA primers. First-strand cDNA was generated from the total RNA using M-MLV Reverse Transcriptase, RNase [H-] (Promega, Madison, WI, USA). cDNA synthesis was tested by PCR using *Actin2* (*ACT2*) primers. Quantitative real-time PCR was performed with three biological and at least two technical replicates with SybrGreen using the first-strand cDNA as a template on an Agilent (Santa Clara, CA, USA) Mx3000P QPCR System, using *ACT2* (At3g18780) as the housekeeping gene. The thermal profile was: 1 cycle at 95 °C for 5 min, 40 cycles at 95 °C for 15 s, 60 °C for 30 s, 72 °C for 30 s. After amplification, a dissociation curve analysis (from 60 °C to 95 °C where the temperature is increased by 0.5 °C s^−1^) was carried out to test primer specificity and check for the absence of primer dimers. Gene expression was calculated according to the 2-ΔΔCT (2-ΔΔCt) method [42]. All primers used for qRT-PCR are listed in Supplementary Table S1.

### 4.7. Analysis of Endogenous Hormones

Approximately 3 g of rosettes were extracted and purified as previously described [43,44,45,46]. The material was homogenized in 80% (*v*/*v*) cold methanol (1:5, *w*/*v*) using a microdevice. Deuterated GAs ([17,17-^2^H_2_]-GA_9_, [17,17-^2^H_2_]-GA_4_, [17,17-^2^H_2_]-GA_34_, [17,17-^2^H_2_]-GA_7_, [17,17-^2^H_2_]-GA_51_, [17,17-^2^H_2_]-GA_19_, [17,17-^2^H_2_]-GA_20_, [17,17-^2^H_2_]-GA_29_, [17,17-^2^H_2_]-GA_1_, [17,17-^2^H_2_]-GA_8_, [17,17-2H2]-GA_3_) and ^13^C_6_-IAA, 75 ng each (OlChemlm Ltd., Olomouc, Czech Republic) were added as internal standards to account for purification losses.

Methanol was evaporated at 35 °C under a vacuum and then the pH was adjusted to 2.8 and the aqueous phase was extracted with ethyl acetate. Extracts were dried and resuspended in 0.3–0.5 mL of water containing 0.01% acetic acid and 10% methanol. Samples were analysed by HPLC analysis using a Kontron instrument (Munich, Germany) with a UV detector (operating at 214 nm). The flow rate used to separate samples was 1 mL min−1 over 150 × 4.6 mm ID ODS Hypersil (Thermo scientific, Waltham, MA, USA) with a particle size of 5 μm. Elution was isocratic, at 10% MeOH, for 4 min. Then, a double gradient from 10% to 30% MeOH was applied over 20 min, followed by 30% to 100% MeOH over 40 min. Fractions that corresponded to elution volumes of standard GAs were collected separately. Fractions were then separated on a 250 × 4.6 mm ID, Nucleosil 100-5 N(CH3)2 column (Macherey-Nagel, Dueren, Germany) and eluted isocratically using 100% methanol that contained 0.01% acetic acid and a flow rate of 1 mL min-1. Fractions corresponding to elution volumes of standard GAs were collected, and then they were dried and silylated with N,O bis (trimethylsilyl) trifluoroacetamide containing 1% trimethylchlorosilane (Pierce, Rockford, IL, USA) for 1 h at 70 °C.

A Saturn 2200 quadrupole ion trap mass spectrometer coupled to a CP-3800 gas chromatograph (Varian Analytical Instruments, Walnut Creek, CA, USA) equipped with a MEGA 1MS capillary column (30 m × 0.25 mm i.d., 0.25 µm film thickness) was used for chromatography tandem mass spectrometry (GC-MS/MS) analysis [45]. Dried and air-free helium was used as the carrier gas, using a linear speed of 60 cm s^−1^. Oven temperature was kept at 80 °C for 2 min, then increased to 300 °C at 20 °C min-1. The injector and transfer line were at 250 °C and the ion source temperature was at 200 °C [46]. Full-scan mass spectra were obtained using EI+ mode, an emission current of 10 µA and an axial modulation of 4 V. Data acquisition was performed from 150 to 600 Da at a speed of 1.4 scan s^−1^. Data were the means of three biological replicates. Gibberellins were identified by comparison to standards using full mass spectra. Quantification was performed with reference to standard plots of concentration ratios versus ion ratios, obtained by analysing known mixtures of unlabelled and labelled GAs.

## Supplementary Materials

Supplementary materials can be found at https://www.mdpi.com/1422-0067/21/20/7789/s1. Table S1: Sequence of primers used for PCR; Figure S1. *AtCuAOδ* T-DNA insertion lines. (a) Locations of the two T-DNA insertions on *AtCuAOδ* in the two lines used (C#4 and BIS#4); (b) PCR showing confirmation of the insertion positions and genotype of the mutant plants. Figure S2: *AtCuAOδ* over-expression lines. (a) Schematic representation of the construct used for generating the transgenic lines; (b) Western blot analysis of crude extract from line 27 over-expressing *AtCuAOδ*; (c) real-time PCR analysis of over-expressor line P27 compared to WT. Figure S3: Phenotypic changes in *AtCuAOδ* mutant and over-expression lines. Figure S4: Germination and leaf emergence in over-expressor lines of *AtCuAOδ.*
