# Supplementary material for: Mutation of Arabidopsis Copper-Containing Amine Oxidase Gene AtCuAOδ Alters Polyamines, Reduces Gibberellin Content and Affects Development
Source: Int J Mol Sci. 2020 Oct 21;21(20):7789. doi: 10.3390/ijms21207789 (PMC7589035; doi:10.3390/ijms21207789)
Supplement: Supplementary file 1 [file ijms-21-07789-s001.zip › ijms-964977-supplementary.pptx]

## Slide 1
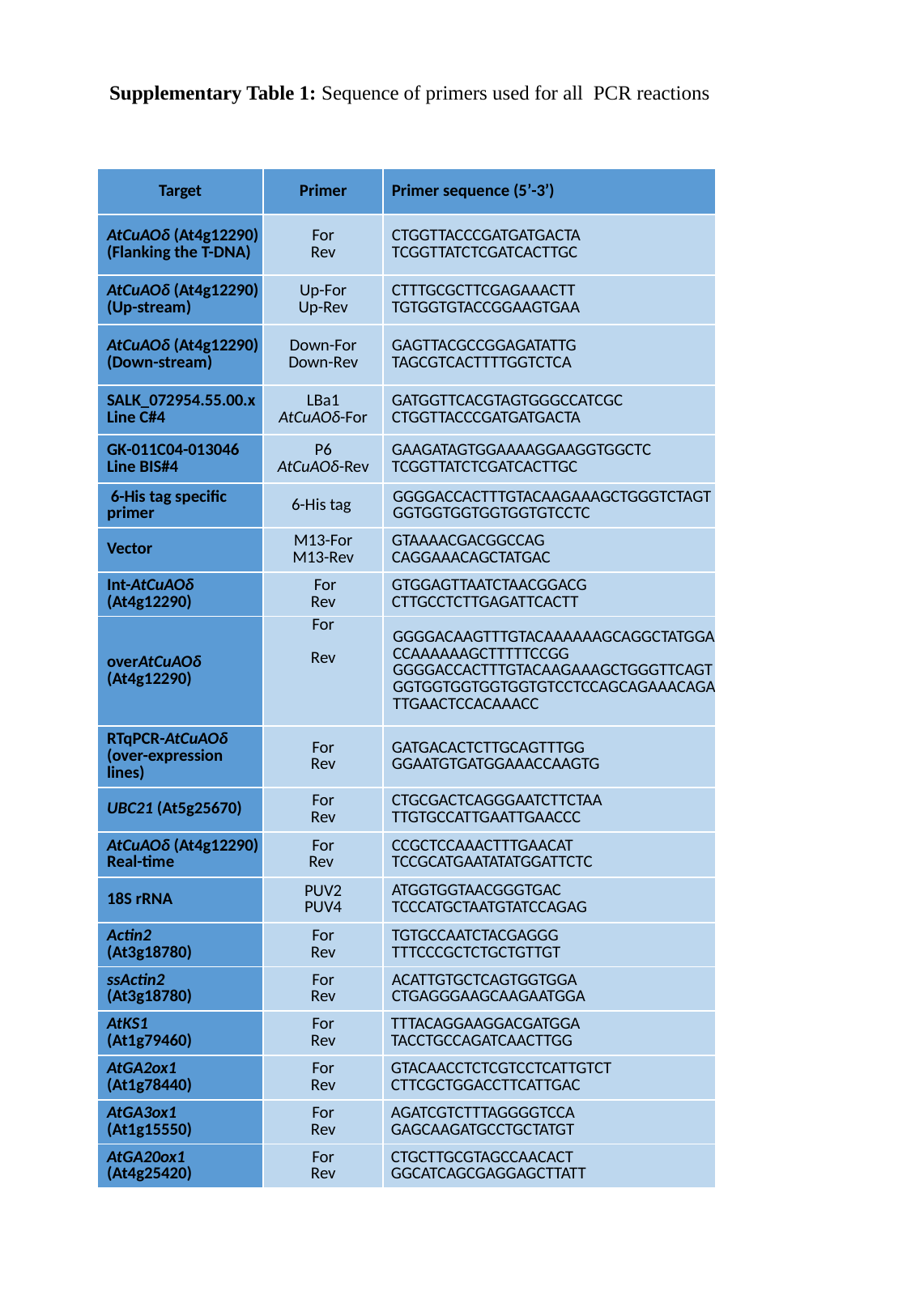

Supplementary Table 1: Sequence of primers used for all PCR reactions
| Target | Primer | Primer sequence (5’-3’) |
| --- | --- | --- |
| AtCuAOδ (At4g12290) (Flanking the T-DNA) | For Rev | CTGGTTACCCGATGATGACTA TCGGTTATCTCGATCACTTGC |
| AtCuAOδ (At4g12290) (Up-stream) | Up-For Up-Rev | CTTTGCGCTTCGAGAAACTT TGTGGTGTACCGGAAGTGAA |
| AtCuAOδ (At4g12290) (Down-stream) | Down-For Down-Rev | GAGTTACGCCGGAGATATTG TAGCGTCACTTTTGGTCTCA |
| SALK\_072954.55.00.x Line C#4 | LBa1 AtCuAOδ-For | GATGGTTCACGTAGTGGGCCATCGC CTGGTTACCCGATGATGACTA |
| GK-011C04-013046 Line BIS#4 | P6 AtCuAOδ-Rev | GAAGATAGTGGAAAAGGAAGGTGGCTC TCGGTTATCTCGATCACTTGC |
| 6-His tag specific primer | 6-His tag | GGGGACCACTTTGTACAAGAAAGCTGGGTCTAGTGGTGGTGGTGGTGGTGTCCTC |
| Vector | M13-For M13-Rev | GTAAAACGACGGCCAG CAGGAAACAGCTATGAC |
| Int-AtCuAOδ (At4g12290) | For Rev | GTGGAGTTAATCTAACGGACG CTTGCCTCTTGAGATTCACTT |
| overAtCuAOδ (At4g12290) | For Rev | GGGGACAAGTTTGTACAAAAAAGCAGGCTATGGACCAAAAAAGCTTTTTCCGG GGGGACCACTTTGTACAAGAAAGCTGGGTTCAGTGGTGGTGGTGGTGGTGTCCTCCAGCAGAAACAGATTGAACTCCACAAACC |
| RTqPCR-AtCuAOδ (over-expression lines) | For Rev | GATGACACTCTTGCAGTTTGG GGAATGTGATGGAAACCAAGTG |
| UBC21 (At5g25670) | For Rev | CTGCGACTCAGGGAATCTTCTAA TTGTGCCATTGAATTGAACCC |
| AtCuAOδ (At4g12290) Real-time | For Rev | CCGCTCCAAACTTTGAACAT TCCGCATGAATATATGGATTCTC |
| 18S rRNA | PUV2 PUV4 | ATGGTGGTAACGGGTGAC TCCCATGCTAATGTATCCAGAG |
| Actin2 (At3g18780) | For Rev | TGTGCCAATCTACGAGGG TTTCCCGCTCTGCTGTTGT |
| ssActin2 (At3g18780) | For Rev | ACATTGTGCTCAGTGGTGGA CTGAGGGAAGCAAGAATGGA |
| AtKS1 (At1g79460) | For Rev | TTTACAGGAAGGACGATGGA TACCTGCCAGATCAACTTGG |
| AtGA2ox1 (At1g78440) | For Rev | GTACAACCTCTCGTCCTCATTGTCT CTTCGCTGGACCTTCATTGAC |
| AtGA3ox1 (At1g15550) | For Rev | AGATCGTCTTTAGGGGTCCA GAGCAAGATGCCTGCTATGT |
| AtGA20ox1 (At4g25420) | For Rev | CTGCTTGCGTAGCCAACACT GGCATCAGCGAGGAGCTTATT |

## Slide 2
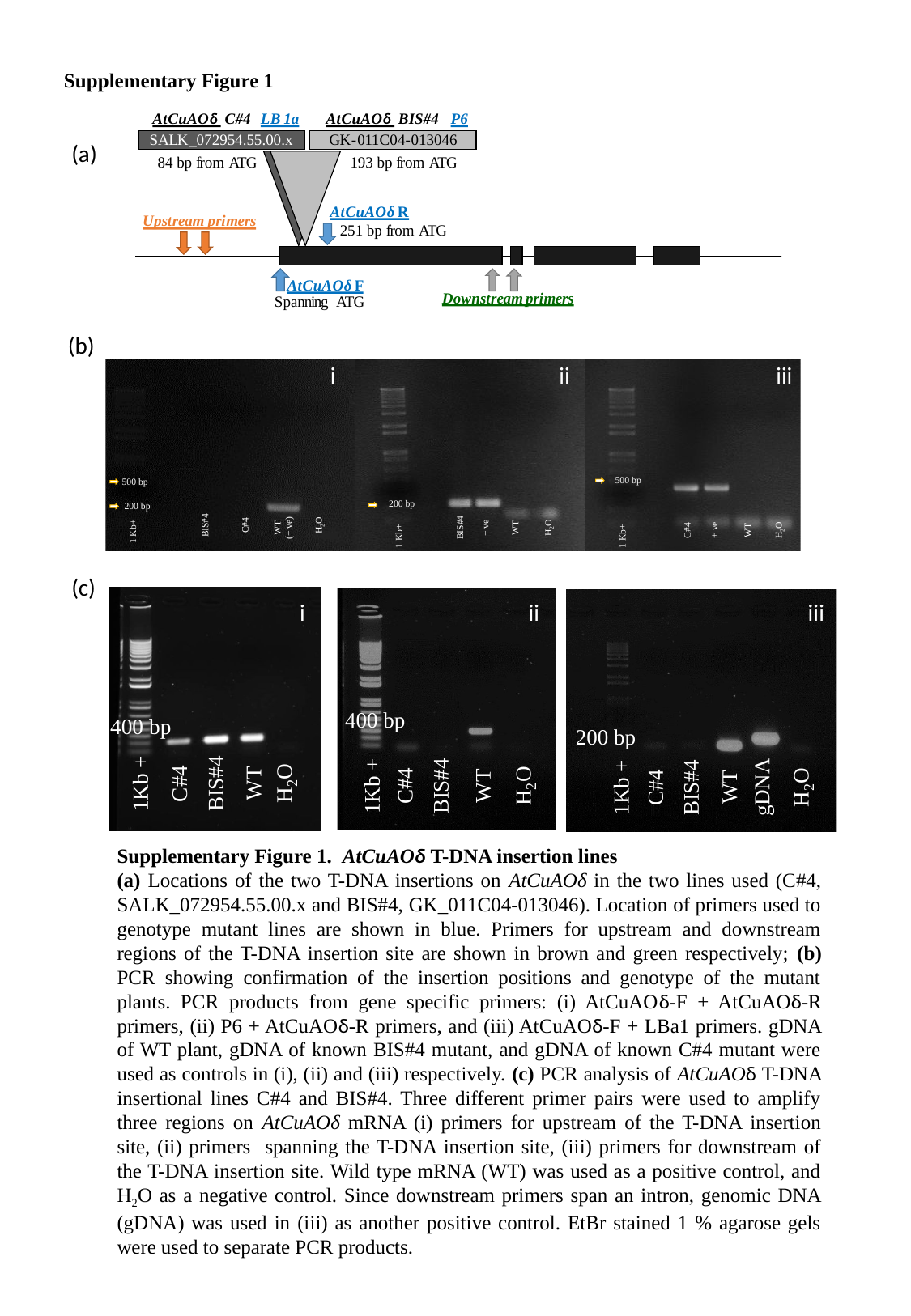

Supplementary Figure 1
(a)
(b)
iii
i
ii
(c)
iii
i
ii
Supplementary Figure 1. AtCuAOδ T-DNA insertion lines
(a) Locations of the two T-DNA insertions on AtCuAOδ in the two lines used (C#4, SALK_072954.55.00.x and BIS#4, GK_011C04-013046). Location of primers used to genotype mutant lines are shown in blue. Primers for upstream and downstream regions of the T-DNA insertion site are shown in brown and green respectively; (b) PCR showing confirmation of the insertion positions and genotype of the mutant plants. PCR products from gene specific primers: (i) AtCuAOδ-F + AtCuAOδ-R primers, (ii) P6 + AtCuAOδ-R primers, and (iii) AtCuAOδ-F + LBa1 primers. gDNA of WT plant, gDNA of known BIS#4 mutant, and gDNA of known C#4 mutant were used as controls in (i), (ii) and (iii) respectively. (c) PCR analysis of AtCuAOδ T-DNA insertional lines C#4 and BIS#4. Three different primer pairs were used to amplify three regions on AtCuAOδ mRNA (i) primers for upstream of the T-DNA insertion site, (ii) primers spanning the T-DNA insertion site, (iii) primers for downstream of the T-DNA insertion site. Wild type mRNA (WT) was used as a positive control, and H2O as a negative control. Since downstream primers span an intron, genomic DNA (gDNA) was used in (iii) as another positive control. EtBr stained 1 % agarose gels were used to separate PCR products.

## Slide 3
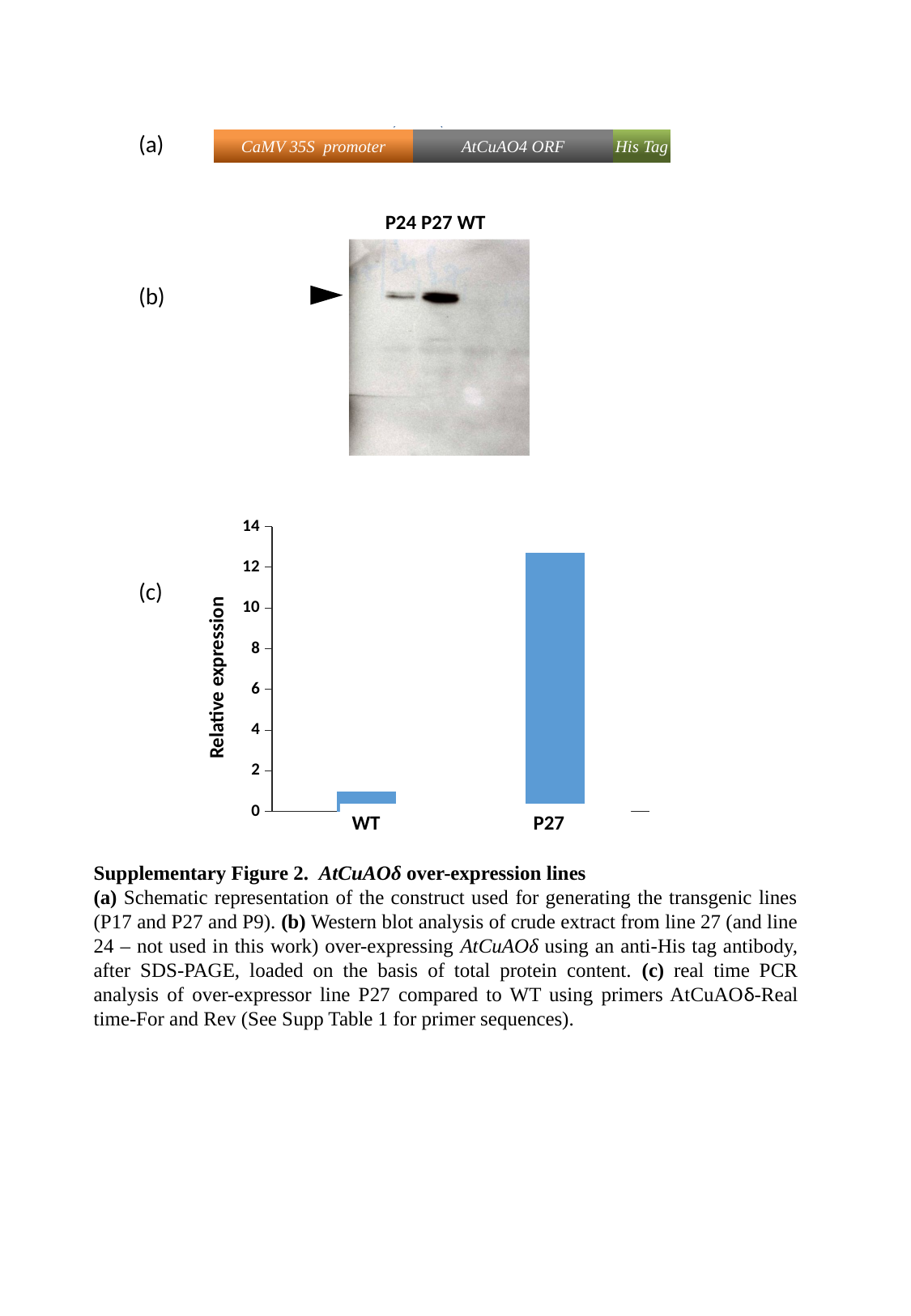

(a)
	 P24 P27 WT
(b)
### Chart
| Category | |
|---|---|
| CNT | 1.0 |
| P27 | 12.7285837400787 |(c)
Relative expression
WT P27
Supplementary Figure 2. AtCuAOδ over-expression lines
(a) Schematic representation of the construct used for generating the transgenic lines (P17 and P27 and P9). (b) Western blot analysis of crude extract from line 27 (and line 24 – not used in this work) over-expressing AtCuAOδ using an anti-His tag antibody, after SDS-PAGE, loaded on the basis of total protein content. (c) real time PCR analysis of over-expressor line P27 compared to WT using primers AtCuAOδ-Real time-For and Rev (See Supp Table 1 for primer sequences).

## Slide 4
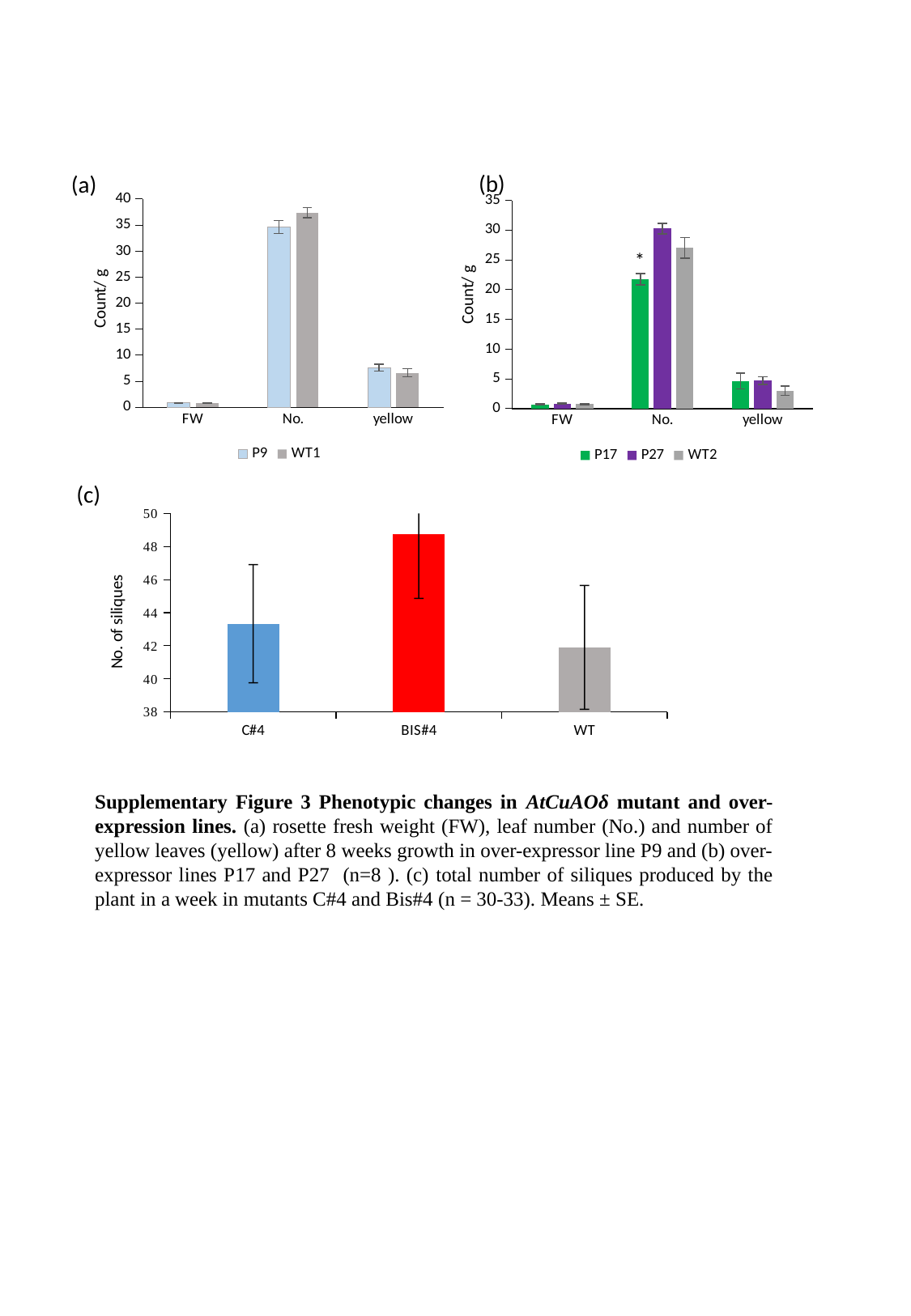

(b)
(a)
### Chart
| Category | P9 | WT1 |
|---|---|---|
| FW | 0.8781 | 0.8408625000000001 |
| No. | 34.625 | 37.375 |
| yellow | 7.625 | 6.625 |
### Chart
| Category | P17 | P27 | WT2 |
|---|---|---|---|
| FW | 0.6862750000000001 | 0.8255875000000001 | 0.737925 |
| No. | 21.75 | 30.25 | 27.0 |
| yellow | 4.625 | 4.714285714285714 | 3.0 |*
Count/ g
Count/ g
(c)
### Chart
| Category | No of siliques produced in a week |
|---|---|
| C#4 | 43.333333333333336 |
| BIS#4 | 48.774193548387096 |
| WT | 41.9 |Supplementary Figure 3 Phenotypic changes in AtCuAOδ mutant and over-expression lines. (a) rosette fresh weight (FW), leaf number (No.) and number of yellow leaves (yellow) after 8 weeks growth in over-expressor line P9 and (b) over-expressor lines P17 and P27 (n=8 ). (c) total number of siliques produced by the plant in a week in mutants C#4 and Bis#4 (n = 30-33). Means ± SE.

## Slide 5
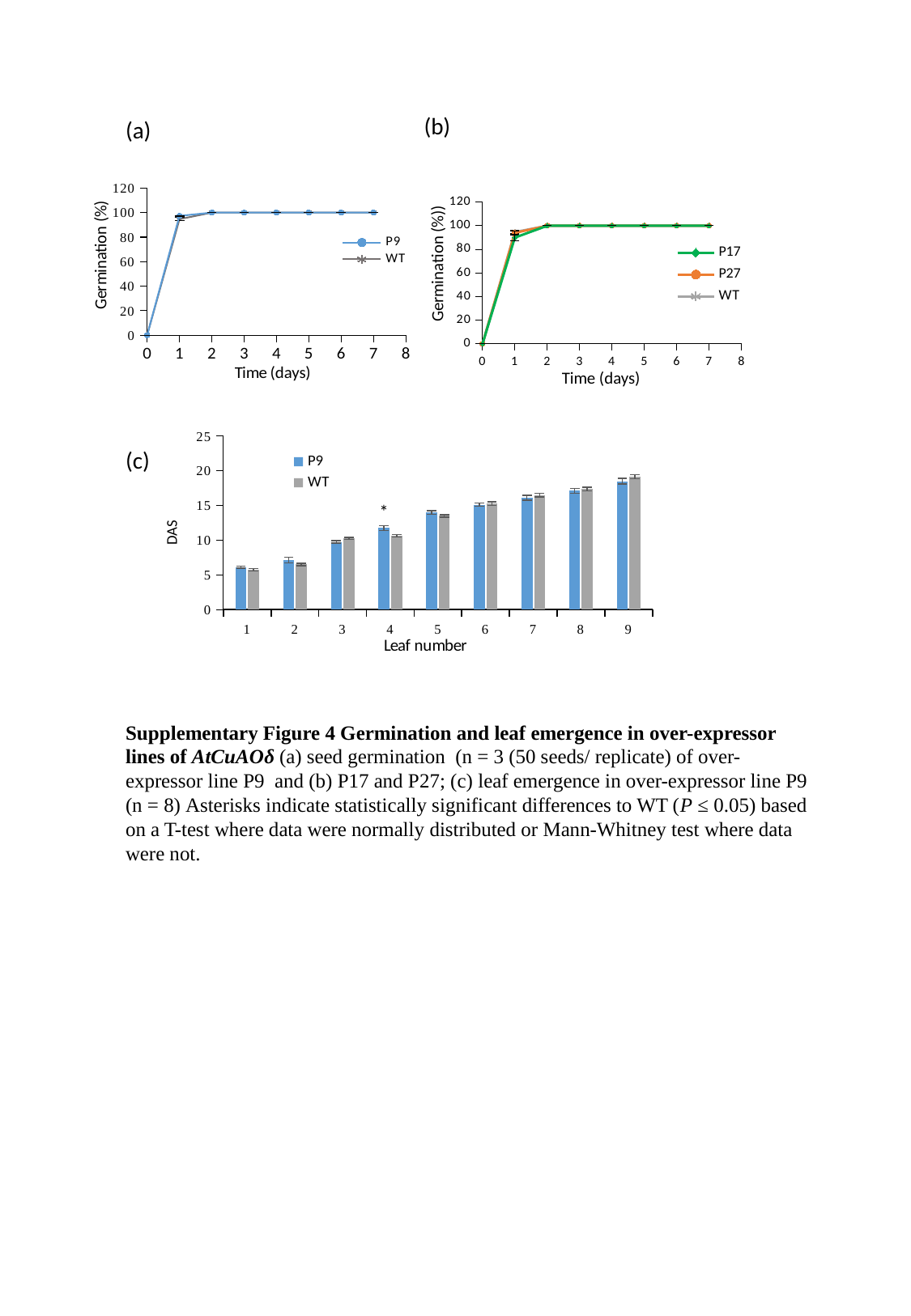

(b)
(a)
### Chart
| Category | P9 | WT |
|---|---|---|
### Chart
| Category | P17 | P27 | WT |
|---|---|---|---|Germination (%))
Time (days)
### Chart
| Category | P9 | WT |
|---|---|---|
| 1 | 6.125 | 5.75 |
| 2 | 7.125 | 6.5 |
| 3 | 9.75 | 10.25 |
| 4 | 11.75 | 10.625 |
| 5 | 14.0 | 13.5 |
| 6 | 15.125 | 15.25 |
| 7 | 16.125 | 16.5 |
| 8 | 17.125 | 17.375 |
| 9 | 18.5 | 19.125 |(c)
Supplementary Figure 4 Germination and leaf emergence in over-expressor lines of AtCuAOδ (a) seed germination (n = 3 (50 seeds/ replicate) of over-expressor line P9 and (b) P17 and P27; (c) leaf emergence in over-expressor line P9 (n = 8) Asterisks indicate statistically significant differences to WT (P ≤ 0.05) based on a T-test where data were normally distributed or Mann-Whitney test where data were not.
